# Mutational Landscape of Pirin and Elucidation of the Impact of Most Detrimental Missense Variants That Accelerate the Breast Cancer Pathways: A Computational Modelling Study

**DOI:** 10.3389/fmolb.2021.692835

**Published:** 2021-06-28

**Authors:** Muhammad Suleman, Muhammad Tahir ul Qamar, Shoaib Saleem, Sajjad Ahmad, Syed Shujait Ali, Haji Khan, Fazal Akbar, Wajid Khan, Adel Alblihy, Faris Alrumaihi, Muhammad Waseem, Khaled S. Allemailem

**Affiliations:** ^1^ Center for Biotechnology and Microbiology, University of Swat, Swat, Pakistan; ^2^ College of Life Science and Technology, Guangxi University, Nanning, China; ^3^ National Center for Bioinformatics, Quaid-i-Azam University, Islamabad, Pakistan; ^4^ Department of Health and Biological Sciences, Abasyn University, Peshawar, Pakistan; ^5^ Medical Center, King Fahad Security College (KFSC), Riyadh, Saudi Arabia; ^6^ Department of Medical Laboratories, College of Applied Medical Sciences, Qassim University, Buraydah, Saudi Arabia; ^7^ Faculty of Rehabilitation and Allied Health Science, Riphah International University, Islamabad, Pakistan

**Keywords:** nsSNPs, PIR protein, breast cancer, MD simulations, PIR

## Abstract

Pirin (*PIR*) protein is highly conserved in both prokaryotic and eukaryotic organisms. Recently, it has been identified that *PIR* positively regulates breast cancer cell proliferation, xenograft tumor formation, and metastasis, through an enforced transition of G1/S phase of the cell cycle by upregulation of E2F1 expression at the transcriptional level. Keeping in view the importance of *PIR* in many crucial cellular processes in humans, we used a variety of computational tools to identify non-synonymous single-nucleotide polymorphisms (SNPs) in the *PIR* gene that are highly deleterious for the structure and function of *PIR* protein. Out of 173 SNPs identified in the protein, 119 are non-synonymous, and by consensus, 24 mutations were confirmed to be deleterious in nature. Mutations such as V257A, I28T, and I264S were unveiled as highly destabilizing due to a significant stability fold change on the protein structure. This observation was further established through molecular dynamics (MD) simulation that demonstrated the role of the mutation in protein structure destability and affecting its internal dynamics. The findings of this study are believed to open doors to investigate the biological relevance of the mutations and drugability potential of the protein.

## Introduction

Pirin (*PIR*) protein is considered highly conserved in both prokaryotic and eukaryotic organisms; however, its biological functions are poorly described (Dunwell et al., 2001; Pang et al., 2004). Pirin is reported as a biomarker in breast cancer, which is abnormal and irregular proliferation of cells associated with inappropriate stimulation of pathways involved in signal transduction (Feitelson et al., 2015; Riaz et al., 2017; Chang et al., 2019). The crystal structure of the human *PIR* gene revealed its quercetinase (acts on quercetin flavonoid) and regulatory functions in many cellular pathways like an inhibitor of protein kinase, antioxidant as well as putative transcriptional co-factor (Chen et al., 2004; Wendler et al., 1997). Previous studies reported the overexpression of *PIR* in different neoplastic transformation and its role in the enhancement of tumor formation due to inducing the expression of Bcl3 by forming the ternary complex with proto-oncogenes Bcl3 and NF-kB (Zhu et al., 2003; Massoumi et al., 2009). Recently, it has been identified that *PIR* positively regulates breast cancer cell proliferation, xenograft tumor formation, and metastasis, through an enforced transition of G1/S phase of the cell cycle by upregulation of E2F1 expression at the transcriptional level (Suleman et al., 2019). It was a significant breakthrough in unveiling the hidden function of *PIR* in the field of cancer.

The most frequently occurring genetic variations are single-nucleotide polymorphisms (SNPs), which disturb both coding and non-coding regions of DNA. SNPs occur in every 200–300 bp in the human genome and consist of about 90% of the total genetic variations in the human genome. The nsSNPs (non-synonymous single-nucleotide polymorphisms) are the various mutations that occur in exonic regions and change the protein sequence, structure, and normal function by triggering modifications in the mechanism of transcription and translation.

Recently, various *in silico* computational tools, methods, and approaches were adopted to investigate the possible role of non-synonymous variation in protein structure and function efficiently and accurately (Kumar et al., 2009; Wadood et al., 2017; Muneer et al., 2019). These methods are of great interest to decipher important molecular mechanisms from protein–protein binding to drug development (Khan et al., 2020a; Khan et al., 2020b; Khan et al., 2020c; Khan et al., 2020d; Khan et al., 2021a; Khan et al., 2021b; Khan et al., 2021c). So far, a total of 173 SNPs comprising 119 missense mutations have been described in the human *PIR* gene and deposited to the database gnomAD (Karczewski et al., 2020).

The *PIR* gene is very polymorphic and is involved in tumorigenesis; however, at this stage, we are uncertain about the effects of the reported nsSNPs on protein structure and biological activities. Therefore, in the present study, with the help of various computational approaches, highly deleterious nsSNPs in the *PIR* gene will be identified, which profoundly affect the structure and function of *PIR* protein. This study is the first extensive *in silico* analysis of the *PIR* gene that can narrow down the candidate mutations for further validation and targeting for therapeutic purposes.

## Materials and Methods

### Pirin Sequence and 3D Structure Data Collection

The online public resources were used to retrieve all the available data about the human *PIR* gene. All the experimentally reported single-nucleotide polymorphisms (SNPs) in the *PIR* gene were collected from an online database gnomAD (https://gnomad.broadinstitute.org/) (Karczewski et al., 2020), and the UniProt database (http://www.uniprot.org/) (Magrane, 2011) was used to retrieve the amino acid sequence (UniProt ID: O00625) that encodes for *PIR* protein. The already reported crystal structure (PDB ID: 6N0J) of *PIR* protein was obtained from the Protein Data Bank (http://www.rcsb.org/) (Rose et al., 2010).

## Data Processing

### Prediction of Functional Consequences of Non-Synonymous Single-Nucleotide Polymorphisms

Various online servers such as PredictSNP (Bendl et al., 2014), MAPP (Multivariate Analysis of Protein Polymorphism) (Chao et al., 2008), PhD-SNP (Predictor of human Deleterious Single Nucleotide Polymorphisms) (Capriotti and Fariselli, 2017), PolyPhen-2 (Polymorphism Phenotyping version 2) (Adzhubei et al., 2013), SIFT (Sorting Intolerant from Tolerant), SNAP (screening for non-acceptable polymorphisms) (Bromberg et al., 2008), and PANTHER (Protein ANalysis THrough Evolutionary Relationships) (Mi et al., 2019) were used to predict the functional effect of nsSNPs. The deleterious nsSNPs, as suggested by all servers, were selected for further analysis. PredictSNP (https://loschmidt.chemi.muni.cz/predictsnp1/) executes prediction with diverse tools and provides a more authentic and accurate substitute for the predictions provided by the individual integrated tool. The predictions by tools in the PredictSNP server are enhanced by experimental annotations from two databases (24). MAPP (http://mendel.stanford.edu/SidowLab/downloads/MAPP/) predicts the effect of all possible SNPs on the function of the protein by considering the physiochemical deviation present in a column of aligned protein sequence (Stone and Sidow, 2005). PhD-SNP (http://snps.biofold.org/phd- snp/phd-snp.html) predicts and divides nsSNPs into disease-related and neutral polymorphisms according to the score ranging from 0 to 1. This server considers SNPs as a disease associated with a score more than 0.5 by using a related program algorithm. The outputs of PhD-SNP depend on frequencies of wild and mutant residues, the conservation index of SNP position, and a number of sequences aligned (Capriotti et al., 2006). PolyPhen-2 (http://genetics.bwh.harvard.edu/pp2) predicts the effect of amino acid variation on protein structure and function. The PolyPhen output is represented with a score that ranges from 0 to 1. This online tool considers non-synonymous SNPs as deleterious, having a higher mutation score, while zero scores indicate no effect of amino acid substitution on protein function (Adzhubei et al., 2010). SIFT (http://sift.bii.a-star.edu.sg) is a program that predicts the effect of amino acid substitution on protein functions. The principles of SIFT predictions depend on the physicochemical properties of protein sequence and its homologies. SIFT classifies its output as deleterious or neutral according to the score ranging from 0 to 1 (0–0.05 as deleterious and 0.05–1 as neutral). (Sim et al., 2012). SNAP (https://rostlab.org/services/snap) is a neural network–based prediction server that identifies the functional effects of amino acid sequence variants. The prediction score ranges from -100 (strongly neutral prediction) to 100 (strong effect prediction), which reflects the likelihood of the single amino acid mutation that may alter the native protein function (Hecht et al., 2015). PANTHER-PSEP (http://www.pantherdb.org/tools/csnpScoreForm.jsp) is an advanced online tool that predicts the non-synonymous mutations that have an important role in human diseases. PANTHER-PSEP uses a correlated but distinctive metric-based evolutionary conservancy. Homologous proteins are used to reconstruct the likely sequences of ancestral proteins at nodes in a phylogenetic tree, and the history of each amino acid can be traced back in time from its current state to estimate how long that state has been preserved in its ancestors.

### Effect of Mutation on Structure Stability and Estimation of Evolutionary Conservation of Non-Synonymous Single-Nucleotide Polymorphisms

To analyze the effect of a mutation on protein stability, we used DynaMut (Rodrigues et al., 2020), a protein stability consensus predictor based on ENCoM’s predicted vibrational entropy changes and the stabilization changes predicted by an mCSM’s graph-based signature method. The degree of the evolutionary conservancy of protein sequence location correlates with the evolutionary degree, which is not the same for all amino acids in the corresponding protein. Positions of amino acids that change slowly are usually known to be conserved sites that are important for the structure and function of a protein.

### Modeling of Wild Type and Variants of Pirin

**FIGURE 1:**
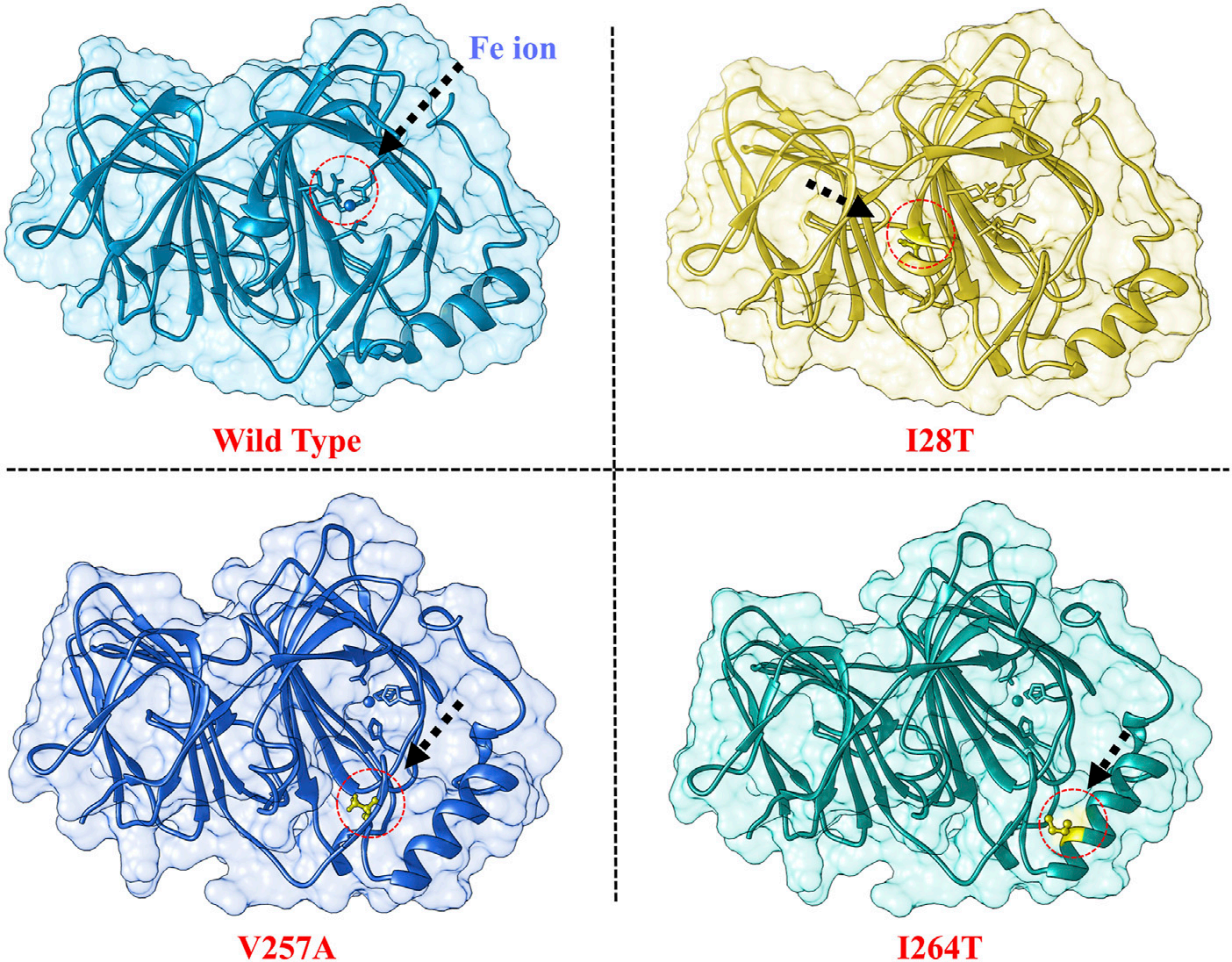
Modeled 3D structures of the WT and I28T, V257A, and I264S mutants. The modeled mutations are encircled to show their position. The Fe ion binding is also shown.

The crystal structure of the *PIR* protein was extracted from the PDB (Entry ID: 6N0J). The protein structure was minimized using Chimera software [(Webb and Sali, 2016),33]. Moreover, the wild type (WT) structure was mutated by each one of the three most deleterious mutants predicted in the previous sections. The three structures of mutant (MT) proteins, such as I28T, V257A, and I264S, were modeled by making a point mutation in the wild-type (WT) protein structure using Chimera software. The WT and three MT structures are shown in Figure 1.

### Molecular Dynamics Simulation

The AMBER18 (Mermelstein et al., 2018) package was used for molecular dynamics simulation to investigate the stability of WT and mutants of pirin (PIR) using the ff14SB force field (Maier et al., 2015). Molecular dynamics (MD) simulation was performed for a total of four systems, including one wild type (WT) and three mutants I28T, V257A, and I264S. For the solvation of each system in a rectangular box water model, TIP3P was used. With the help of counterions, addition neutralization was achieved. A two-step energy minimization procedure: the steepest decent minimizations of 6,000 cycles and conjugate gradient minimization of 3,000 cycles, was applied for minimization of each neutralized system. After minimization, these complexes were heated up to 300 K for 0.2 ns, and then we equilibrated the system with weak restraint and without restraint for 2 ns at 300 K, respectively. The temperature was controlled with a Langevin thermostat (Adzhubei et al., 2010) (26), and the procedure was run for 100 ns. Each simulation was repeated three times. Long-range electrostatic interactions (Bromberg et al., 2008; Sim et al., 2012; Adzhubei et al., 2013; Hecht et al., 2015) were detected with the particle mesh Ewald method (Petersen, 1995) using a cut-off distance of 10.0 Å. The SHAKE method was applied for covalent bond treatment (Mi et al., 2019). The MD simulation production step was performed on the GPU-supported PMEMD code for each system (Glaser et al., 2003; Stone and Sidow, 2005), and the trajectories were analyzed on the CPPTRAJ package in Amber18.

### Principal Component Analysis and Gibbs Free Energy Calculation

Principal component analysis (PCA) was utilized for the calculation of high-amplitude fluctuations within the protein (Berezin et al., 2004; Capriotti et al., 2006). The CPPTRAJ package calculated the covariance matrix based on Cα coordinates. Eigenvectors and eigenvalues were calculated by diagonalizing the covariance matrix. 5,000 snapshots from the trajectory of each system were used to get PCA calculations. Eigenvectors and eigenvalues indicate the direction of motion and mean square fluctuation, respectively. PC1 and PC2 were used for plotting to monitor the motion. The lowest energy stable state was determined by the free energy landscape (FEL) and is indicated by deep valleys on plot, whereas the intermediate state is shown by boundaries between deep valleys (Xu et al., 2017; Adzhubei et al., 2010). In this study, FEL calculations based on PC1 and PC2 were obtained by

ΔG (PC1, PC2) = −KBTln P (PC1, PC2 (1)

where the reaction coordinates are taken by PC1 and PC2, K_B_ denotes the Boltzmann constant, and P (PC1, PC2) shows the probability distribution of the system along with the first two principal components.

## Results and Discussion

### Identification of Deleterious Non-Synonymous Single-Nucleotide Polymorphisms

**FIGURE 2:**
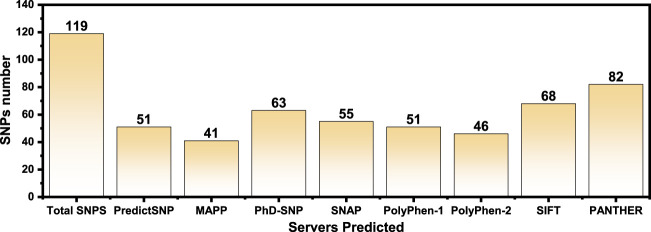
The total number of predicted deleterious SNPs by each server is shown as bars. Each bar represents a specific server, and its predicted deleterious mutations are given on the top.

The online public resources were used to retrieve all the available data of the human PIR gene. According to the information obtained from the online database gnomAD, there were a total of 173 SNPs in the *PIR* protein. Of those, 119 SNPs were identified as non-synonymous. These 119 SNPs were submitted to different online servers for identification of the deleterious mutations. First, the SNPs were submitted to PredictSNP and MAPP servers, and only 51 and 41 SNPs were found as deleterious, respectively. The nsSNPs were then submitted to PhD-SNP and SNAP online tools, and 63 and 55 SNPs were found as deleterious, respectively. The other online servers such as PolyPhen-1, PolyPhen-2, SIFT, and PANTHER analyzed the nsSNPs and predicted that, out of 119 SNPs, only 51, 46, 68, and 80 were deleterious, respectively. All the nsSNPs were selected for further analysis that were predicted as deleterious consistently by all the above online servers as shown in Table 1. The total number of predicted deleterious SNPs by each server is given in Figure 2.

**TABLE 1:** Processing of 119 missense variants by different servers predicted 24 mutations to be deleterious collectively. The predicted score by each server is also shown.

Variant	Predict SNP	MAPP	PhD-SNP	PolyPhen-1	PolyPhen-2	SIFT	SNAP	PANTHER	Outcome
E18G	0.869	0.508	0.676	0.744	0.811	0.792	0.622	0.662	DELETERIOUS
G19A	0.869	0.766	0.773	0.744	0.811	0.792	0.805	0.760	DELETERIOUS
I28T	0.869	0.461	0.676	0.594	0.550	0.792	0.720	0.780	DELETERIOUS
P38L	0.869	0.766	0.858	0.744	0.811	0.792	0.720	0.842	DELETERIOUS
H56Q	0.869	0.765	0.732	0.744	0.811	0.792	0.848	0.842	DELETERIOUS
H58R	0.869	0.919	0.875	0.744	0.811	0.792	0.885	0.874	DELETERIOUS
R59P	0.869	0.841	0.817	0.744	0.811	0.792	0.720	0.714	DELETERIOUS
G60V	0.869	0.913	0.875	0.744	0.811	0.792	0.848	0.874	DELETERIOUS
D77E	0.869	0.819	0.607	0.744	0.811	0.792	0.885	0.744	DELETERIOUS
H81P	0.869	0.656	0.588	0.744	0.453	0.792	0.720	0.718	DELETERIOUS
A95V	0.869	0.760	0.858	0.744	0.811	0.792	0.720	0.780	DELETERIOUS
G98S	0.869	0.573	0.875	0.744	0.811	0.792	0.848	0.744	DELETERIOUS
G98D	0.869	0.877	0.875	0.744	0.811	0.792	0.869	0.874	DELETERIOUS
H101Y	0.869	0.841	0.817	0.744	0.811	0.792	0.885	0.7145	DELETERIOUS
Q115K	0.869	0.856	0.773	0.744	0.811	0.792	0.848	0.744	DELETERIOUS
L116P	0.869	0.774	0.858	0.744	0.811	0.792	0.720	0.744	DELETERIOUS
G179V	0.869	0.751	0.817	0.744	0.562	0.792	0.555	0.744	DELETERIOUS
L220P	0.869	0.765	0.858	0.744	0.675	0.792	0.720	0.760	DELETERIOUS
E248A	0.869	0.718	0.875	0.744	0.647	0.792	0.720	0.686	DELETERIOUS
E248D	0.869	0.656	0.773	0.594	0.562	0.527	0.622	0.734	DELETERIOUS
G254V	0.869	0.842	0.773	0.744	0.811	0.792	0.848	0.780	DELETERIOUS
V257A	0.869	0.819	0.858	0.594	0.675	0.792	0.885	0.766	DELETERIOUS
M258I	0.869	0.559	0.607	0.594	0.600	0.527	0.805	0.698	DELETERIOUS
I264S	0.869	0.806	0.858	0.744	0.634	0.792	0.555	0.842	DELETERIOUS

### Effect of Mutation on Pirin Protein Structure Stability

**FIGURE 3:**
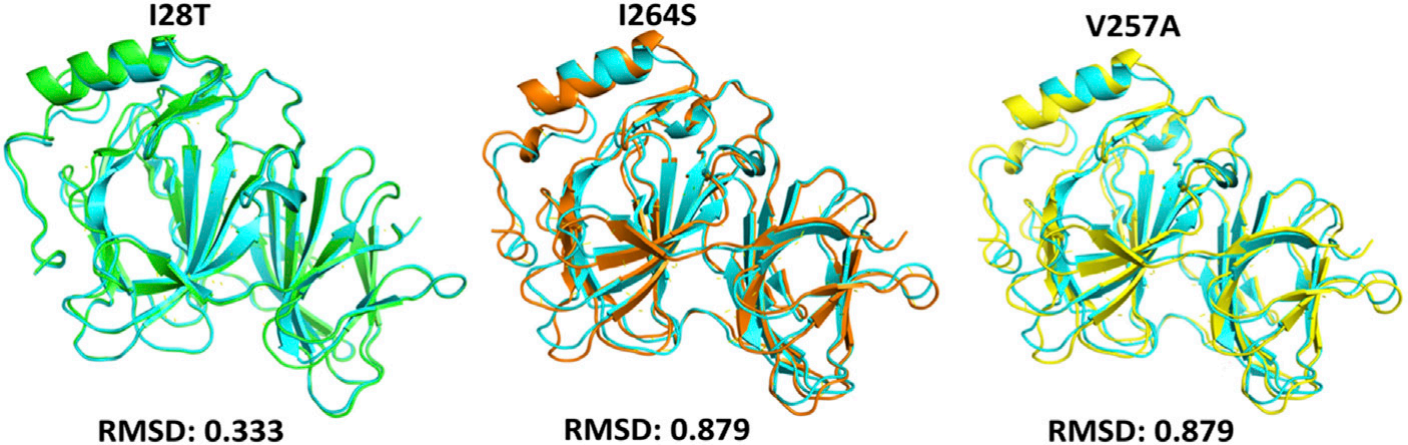
Superimposed structure of WT *PIR* protein (cyan) with mutants I28T (green), I264S (orange), and V257A (yellow). The RMSD of each superimposition was reported to be 0.333 Å (I28T) and 0.879 Å (I264S, V257A).

**FIGURE 4:**
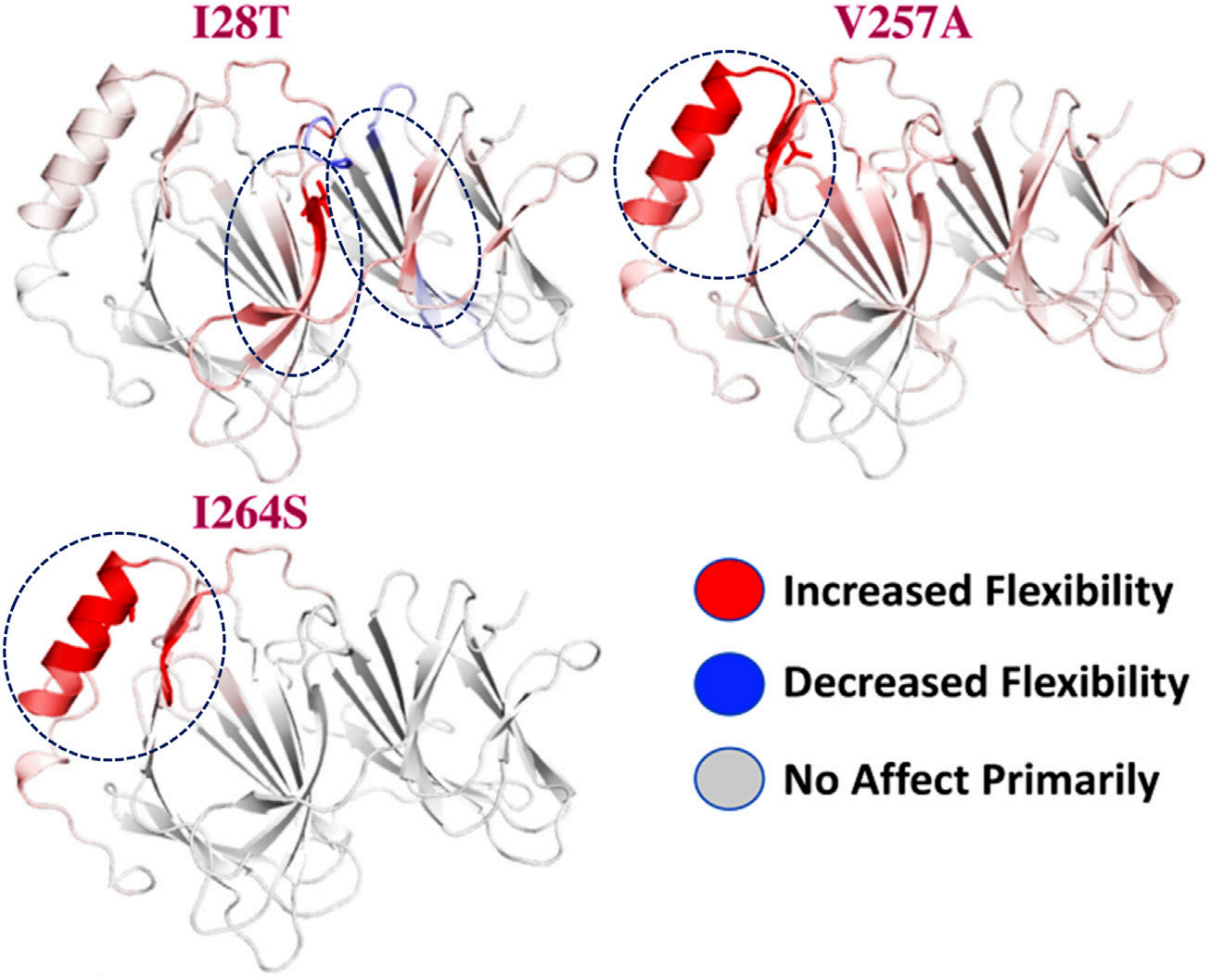
Effect of mutations on structural dynamics flexibility. Changes in flexibility (red) and rigidity (blue) mapped onto the corresponding protein structure are shown.

To calculate the stability changes upon mutations, an online server mCSM was used, which reported the average changes ranging from 0.715 to −2.856 kcal/mol. Mutations, such as V257A with a stability fold change of -2.157 kcal/mol, I28T with a stability fold change of -2.374 kcal/mol, and I264S with a stability fold changes of -2.856 kcal/mol, were found to be highly destabilizing for the *PIR* protein structure. However, the mutation H81P with a stability fold change of 0.715 kcal/mol has the opposite effect (i.e., stability) and does not induce major changes in the protein structure (Table 2). The RMSDs between the WT and the three mutants are shown as a superimposed structure in Figure 3. The highly destabilizing mutations identified by mCSM were analyzed by DynaMut to check the effect of these mutations on the structure flexibility. As shown in Figure 4, the mutations I28T, V257A, and I264S produced higher flexibility in the protein structure. These results are clearly pointing out the importance of these three mutations. These changes in flexibility (red) and rigidity (blue) are mapped onto the corresponding protein structure and presented in Figure 4.

**TABLE 2:** A list of 24 highly deleterious mutations was processed to identify the highly destabilizing mutations.

Index	Mutation	ΔΔG mCSM	Outcome
1	E18G	**−**1.063	Destabilizing
2	G19A	**−**0.265	Destabilizing
**3**	**I28T**	**−2.374**	**Highly destabilizing**
4	P38L	**−**0.839	Destabilizing
5	H56Q	**−**0.777	Destabilizing
6	R59P	**−**1.518	Destabilizing
7	H58R	**−**1.986	Destabilizing
8	G60V	**−**0.63	Destabilizing
9	D77E	**−**0.822	Destabilizing
10	H81P	0.715	Stabilizing
11	A95V	**−**0.937	Destabilizing
12	G98D	**−**1.812	Destabilizing
13	G98S	**−**1.53	Destabilizing
14	H101Y	**−**0.191	Destabilizing
15	Q115K	**−**0.244	Destabilizing
16	L116P	**−**1.237	Destabilizing
17	G179V	**−**0.66	Destabilizing
18	L220P	**−**1.406	Destabilizing
19	E248A	**−**0.764	Destabilizing
20	E248D	**−**0.693	Destabilizing
21	G254V	**−**0.205	Destabilizing
**22**	**V257A**	**−2.157**	**Highly destabilizing**
23	M258I	**−**0.996	Destabilizing
**24**	**I264S**	**−2.856**	**Highly destabilizing**

Based on ΔΔG, the mCSM server predicted I28T, V257A, and I264S as highly destabilizing, while the rest were classified as destabilizing only. Bold are highly destabilizing mutations which were subjected to MD simulation.

### Dynamics Stability and Residual Flexibility of the Wild and Mutant Structures

To estimate the impact of the specific mutant (I28T, V257A, and I264S) and WT, we calculated the RMSD (root mean square deviation) from the MD trajectory. We used 5,000 structures from the MD trajectory as a function of time. In the case of the WT, the RMSD remained stable during the 100 ns simulation time. No significant convergence was observed. The system reached the stability at 1.3 Å. The average RMSD was reported to be 1.25 Å. Overall, the system seems to be stable with no significant convergence during the 100 ns simulation. On the contrary, the I28T mutation showed significant convergence at different intervals. Initially, the structure continued to proceed stably until 20 ns, but after the system faced convergence, the RMSD increased from 1.5 to 2.0 Å.

**FIGURE 5:**
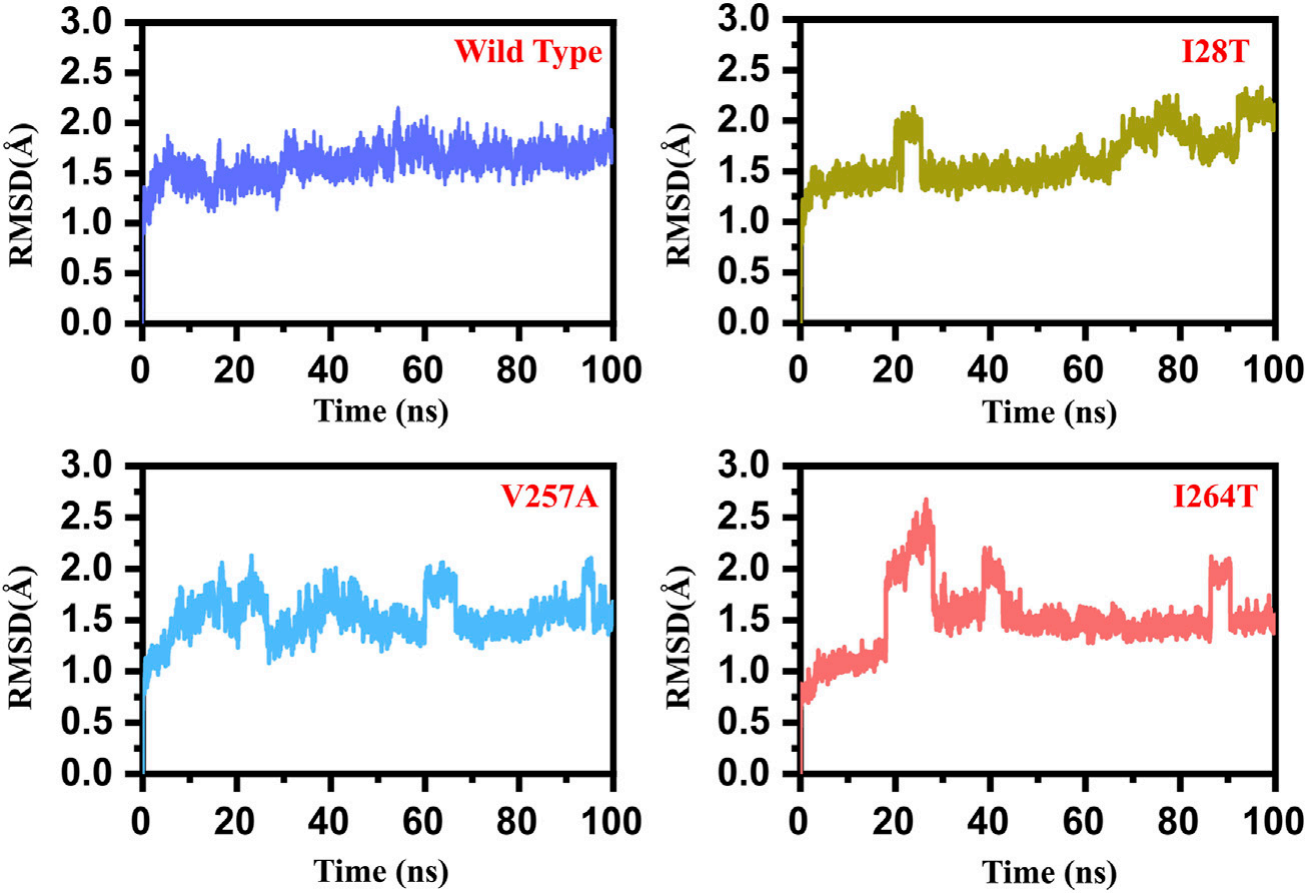
RMSD graph of the WT and mutants (I28T, V257A, and I264S). The *x*-axis shows the time in nanoseconds, while the *y*-axis shows the RMSD in angstrom. **(A)** Wild type; **(B)** I28T; **(C)** V257A; **(D)** I264S.

Afterward, the RMSD decreased and remained uniform until 70 ns, but the structure faced significant perturbation and the RMSD increased again until 100 ns. The major convergence was observed specifically between 75 and 90 ns. The average RMSD (1.8 Å) also remained higher than that in the wild type. This shows that the I28T mutation has caused a significant structural stability shift and needs longer simulation to gain the equilibrium. Furthermore, the V257A mutation also induced significant structural stability changes. The RMSD remained higher during the 100 ns simulation. Initially, the RMSD increased until 1.25 Å and then continued to increase until 20 ns. Afterward, an abrupt decrease was observed at 22 ns, and then again, the RMSD increased. The RMSD between 60 and 80 ns significantly converged, and the average RMSD between 60 and 80 ns was observed to be 2.0 Å. The RMSD then decreased and remained uniform until 95 ns, but then again, the structure converged and the RMSD increased. Hence, the V257A mutation has caused significant structural perturbation, and the stability significantly shifted as compared to that of the wild type. I264S was reported to be the most destabilizing mutation among the list of 24 non-synonymous mutations reported to be deleterious. The results here are uniform with the mCSM server. The mutation has induced significant stability transition and perturbation. Initially until 20 ns, the RMSD remained uniform, but a sudden convergence increased the RMSD up to 2.5 Å. Later on, the RMSD decreased for a short period of time, and then significant convergence was observed between 35 and 40 ns. The RMSD then remained lower and uniform until 85 ns. The structure then faced significant perturbation, and the RMSD increased again up to 2.0 Å. The average RMSD for I264S was reported to be 2.2 Å. Thus, these results signify that the mutations have caused significant structural destability and internal dynamics of the protein. The RMSD graph of the WT and mutants (I28T, V257A, and I264S) is shown in Figure 5. The *x*-axis shows the time in nanoseconds, while the *y*-axis shows the RMSD in angstrom.

**FIGURE 6:**
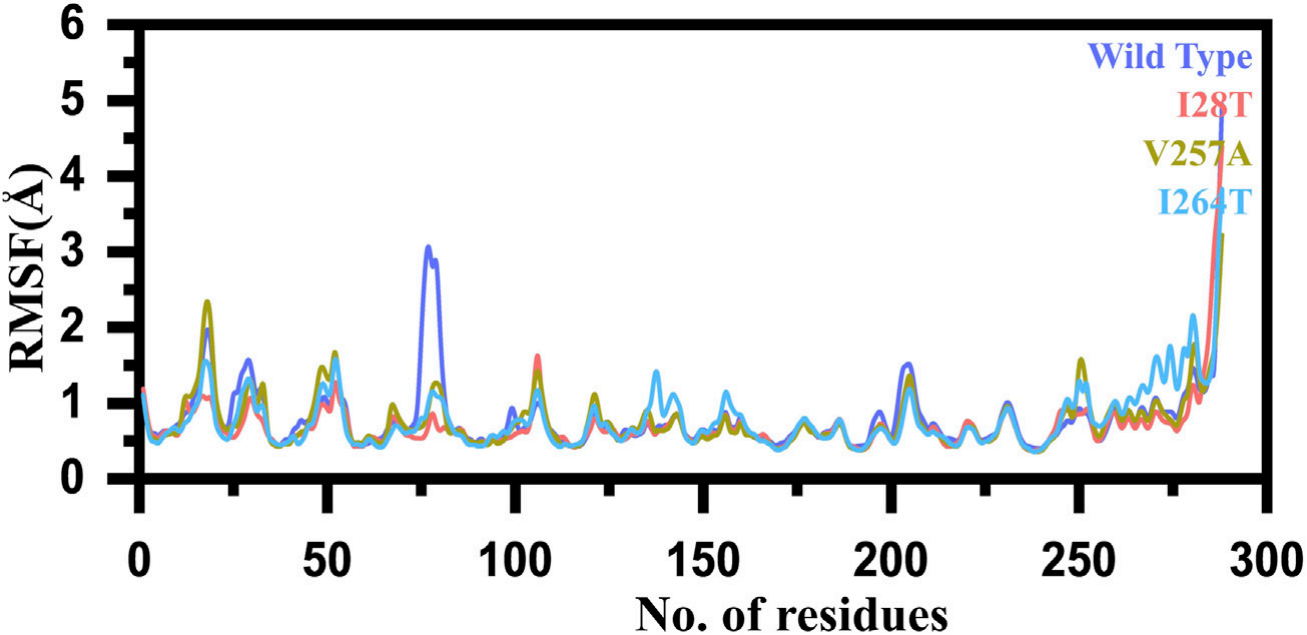
RMSF graph of the WT and mutants (I28T, V257A, and I264S). The *x*-axis shows the number of residues, while the *y*-axis shows the RMSF in angstrom.

Furthermore, to estimate the impact of the mutation on the residual flexibility, we calculated the RMSF (root mean square fluctuation) as a function of residues. Overall, the residual flexibility showed more similar fluctuation except in few regions. In the case of V257A, the region between 15 and 25 showed higher fluctuation than the others. In addition, the region between 72 and 85 in the WT possesses higher fluctuation than the mutants. Thus, this shows that this region is significantly affected by the mutation induction. In the case of I264S, specifically the region between 140 and 150 showed higher fluctuation. Furthermore, this mutation also increased the fluctuation of the region between 250 and 280, thus causing significant internal dynamics fluctuation. These results show that the mutation has affected different regions of the protein to increase or decrease the flexibility. The RMSF graph of the WT and mutants (I28T, V257A, and I264S) is shown in Figure 6. The *x*-axis shows the number of residues, while the *y*-axis shows the RMSF in angstrom.

### Structural Compactness Estimation of the Wild and Mutant Structures

**FIGURE 7:**
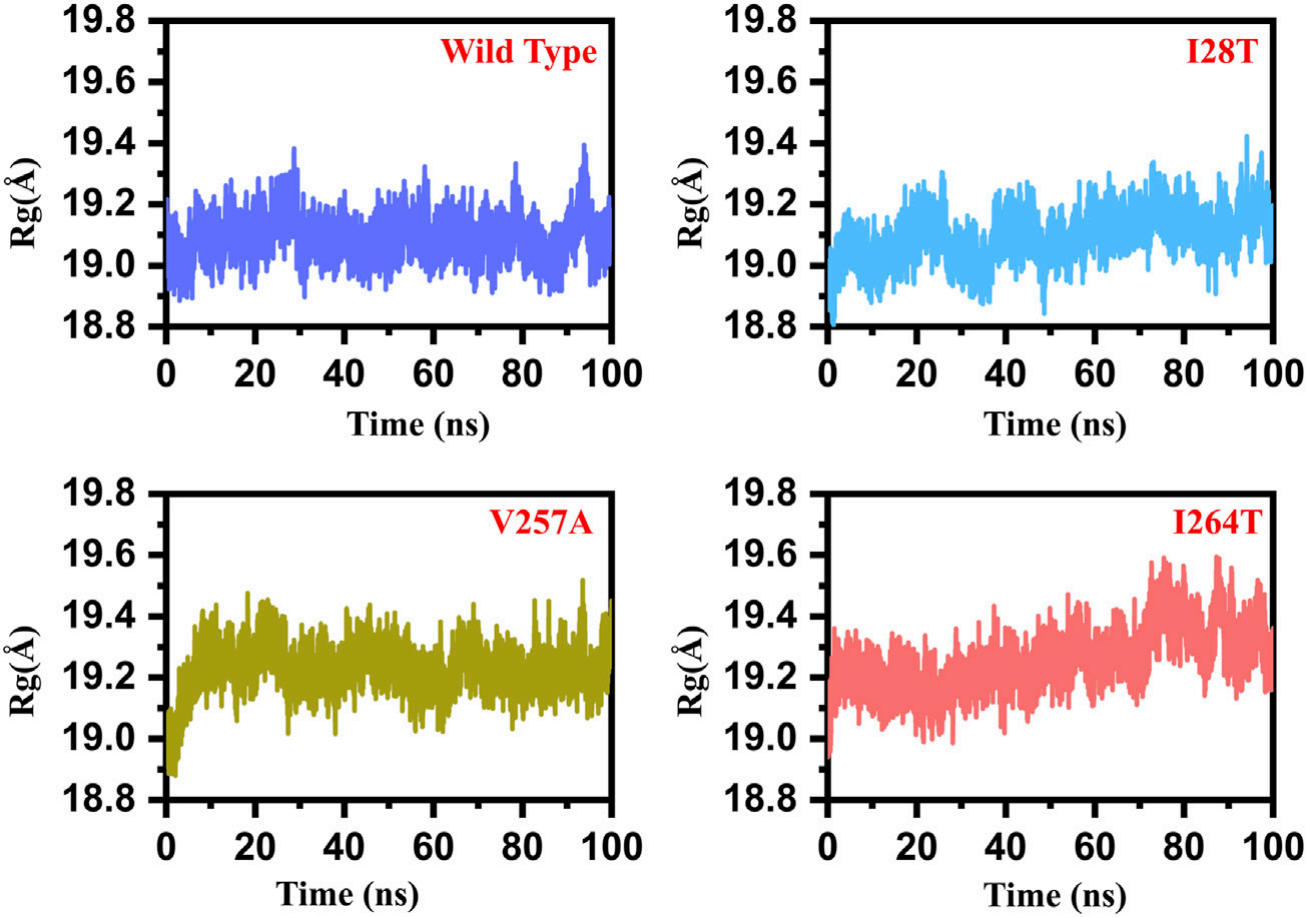
Rg graph of the WT and mutants (I28T, V257A, and I264S). The *x*-axis shows the time in nanoseconds, while the *y*-axis shows the RMSD in angstrom.

In order to calculate the compactness of all the WT and mutant (I28T, V257A, and I264S) systems, R_g_ (radius of gyration) was calculated. The stability of the complexes formed also depended on the compactness of the structure. From Figure 7, it can be easily observed that the average R_g_ value for all the systems is between 19.0 and 19.4 Å. In the case of wild type, the R_g_ value remained uniform until 100 ns. The average value for the WT was observed to be 19.0 Å. In the case of I28T, the system remained relatively less compact than the wild type. The average R_g_ value was reported to be 19.0 Å for the first 52 ns, and then the R_g_ value continued to increase and reached 19.2 Å during the simulation time. In the case of V257A, the R_g_ value remained lower until 5 ns. The R_g_ then continued to increase until 100 ns. The R_g_ value for the rest of 95 ns remained 19.3 Å. The R_g_ for I264S started from 19.2 Å and continued to increase. After reaching 30 ns, the R_g_ value increased to 19.3 Å and increased further. After 70 ns, the R_g_ value further increased to 19.5 Å and continued until 100 ns. These results significantly justify that the mutation has different compactness than the WT during the simulation. The R_g_ graph of the WT and mutants (I28T, V257A, and I264S) is shown Figure 7. The *x*-axis shows the time in nanoseconds, while the *y*-axis shows the R_g_ in angstrom.

### Dimensionality Reduction and Clustering the Protein Motions

**FIGURE 8:**
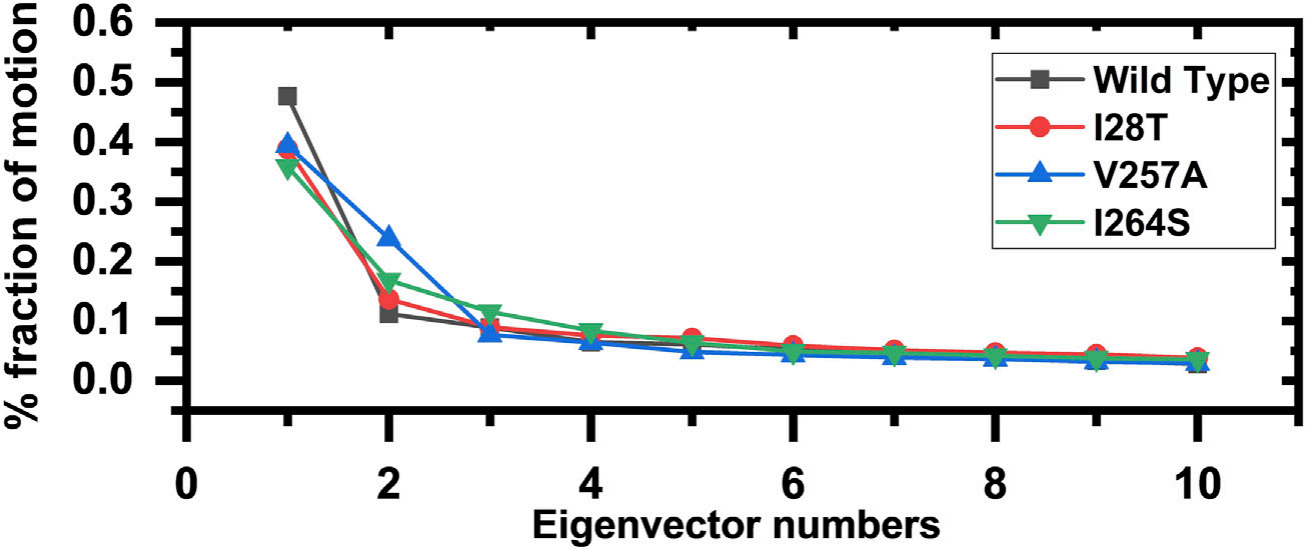
Fractions of the first ten eigenvectors. Using the MD trajectory, the fraction of motions is calculated and given in percentage against the eigenvector numbers.

To describe the protein motion and clustering of the related structural frames, principal component analysis (PCA) was performed. PCA is a statistical approach that incorporates a smaller number of uncorrelated variables called principal components into several correlated variables. The eigenvectors were measured and are provided in Figure 8 to comprehensively explain the effect of the substitution on the protein motion. From the PCs, we can understand the overall and internal motions. In the wild type, the total contributed variance by the first three eigenvectors to the total motions was reported to be 47%, while in the case of I28T, the variance by the three eigenvectors was observed to be 38%, and for V257A, it was observed to be 39%. In the other mutation such as I264S, the variance by the first three eigenvectors was reported to be 35%.

**FIGURE 9:**
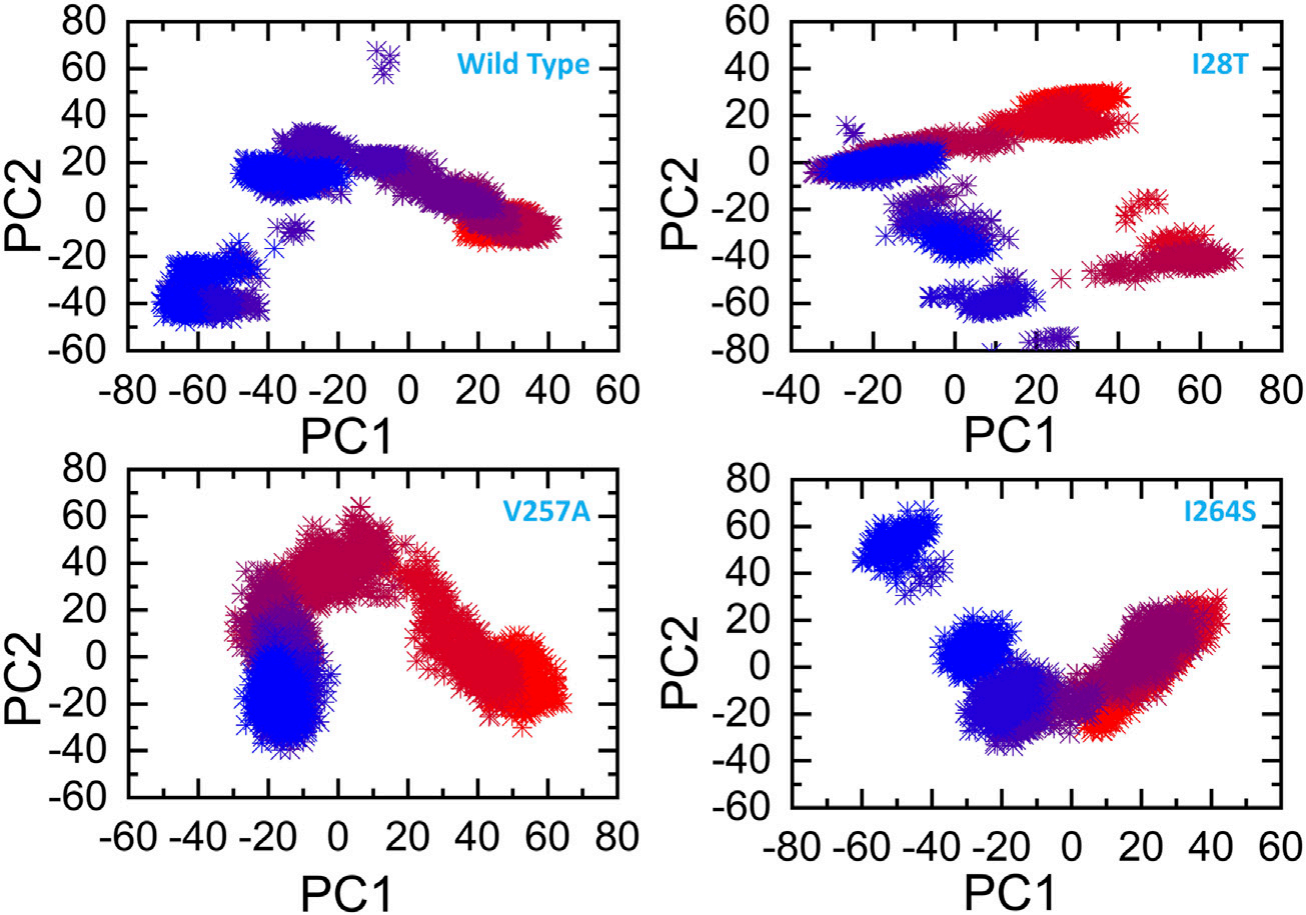
Principal component analysis of all the systems, including the WT and the three mutants. The first two principal components (PC1 and PC2) are used for the projection of motion in the space phase at 300 K.

The other eigenvectors have shown localized or overall motions. Hence, it is confirmed that the mutations have impacted the total trajectory motion and, thus, internal dynamics behavior. To further gain convincible attributes, the first two PCs, i.e., PC1 and PC2, were drawn against each other. Different colors (red to blue) reflect the conformational transition from one to another. Each dot in Figure 9 depicts a single frame of the trajectory. As compared to the WT, the mutant complexes covered a lower region of the phase space except in V257A and I264S. Together, these observations suggest that mutations had a substantial influence on the structure that has contributed to pirin (PIR) destabilization.

**FIGURE 10:**
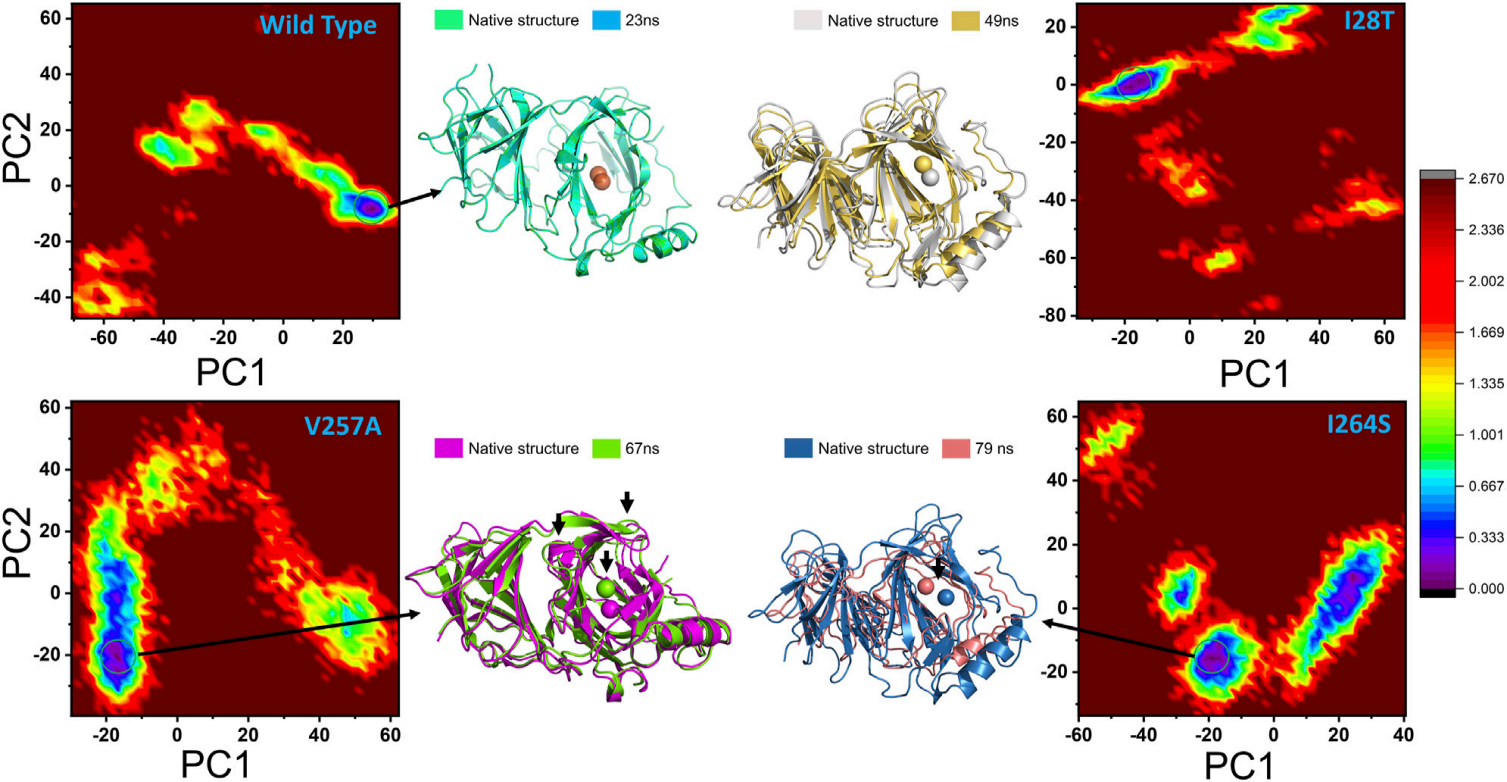
Conformational changes during the molecular dynamics simulation are represented through the FEL. PC1 and PC2 are used to map the energy minima and extract the variations due to a specific mutation.

Conformational changes induced by a particular mutation during the MD simulation were explored through the FEL. PC1 and PC2 were used to map the energy minima and extract the variations due to a specific mutation. In the case of the wild type, the lowest energy minima were reached at 23 ns. Figure 10 shows that, during the simulation, no structural perturbation was experienced in the wild type. On the contrary, in the three mutations, destabilization of the Fe ion was observed. The energy minima separated by subspace in each mutant complex were reached at 49 ns (I28T), 67 ns (V257A), and 79 ns (I264S). In addition, the cavity surrounding the Fe ion also exhibits a dynamic structure in all the mutants. The beta sheet covering the Fe ion from the top and the loop on the alternate side changed their orientations, and an opening–closing switch-like pattern was observed. In addition, the flipping of beta sheets in the mutant complexes was most frequently observed in the mutant complexes. All the FEL graphs of the wild type, I28T, V257A, and I264S are given in Figure 10.

## Conclusion

PIR is an oxidative stress sensor from the functionally diverse superfamily of cupin. This protein is suggested to have biological relevance in cancer development and thus remains a novel research area. Being polymorphic, its oncogenic activity is a hot topic of discussion in the recent past. The work reported herein attempted to use an extensive computational framework to screen all potential mutations of the protein and identify deleterious mutants that could affect protein structure stability and ultimately functionality. The work predicted 119 missense variants by different servers and reported 24 deleterious mutations consistently reported by all available mutation predictor servers. Furthermore, it was highlighted that the three mutations I28T, V257A, and I264S are most destabilizing and confer structure flexibility to the PIR protein. To sum up, the study provides structural basis for each mutation-induced conformational change and disclosed a possible way for the mutations’ role in the progression of Breast Cancer (BC); thus, PIR acts a potential therapeutic target or a biomarker in the future ahead.

## Data Availability

The datasets presented in this study can be found in online repositories. The names of the repository/repositories and accession number(s) can be found in the article/Supplementary Material.
